# Economic Evaluation of Screening Strategy for Latent Tuberculosis Infection (LTBI) in Contacts of Tuberculosis Patients: Systematic Review and Quality Assessment

**DOI:** 10.3390/ijerph192013529

**Published:** 2022-10-19

**Authors:** Panida Yoopetch, Natthakan Chitpim, Jiraphun Jittikoon, Wanvisa Udomsinprasert, Montarat Thavorncharoensap, Sitaporn Youngkong, Naiyana Praditsitthikorn, Surakameth Mahasirimongkol, Usa Chaikledkaew

**Affiliations:** 1 Mahidol University Health Technology Assessment (MUHTA) Graduate Program, Mahidol University, Bangkok 10400, Thailand; 2 Social, Economic, and Administrative Pharmacy Graduate Program, Department of Pharmacy, Faculty of Pharmacy, Mahidol University, Bangkok 10400, Thailand; 3 Department of Biochemistry, Faculty of Pharmacy, Mahidol University, Bangkok 10400, Thailand; 4 Social Administrative Pharmacy Division, Department of Pharmacy, Faculty of Pharmacy, Mahidol University, Bangkok 10400, Thailand; 5 Department of Disease Control, Ministry of Public Health, Nonthaburi 11000, Thailand; 6 Department of Medical Sciences, Medical Genetics Center, Medical Life Sciences Institute, Ministry of Public Health, Nonthaburi 11000, Thailand

**Keywords:** latent tuberculosis infection, interferon gamma release assay, tuberculin skin test, tuberculosis infection

## Abstract

A tuberculin skin test (TST) or interferon-gamma release assay (IGRA) can be used to screen for latent tuberculosis infection (LTBI). Due to its low cost, TST has been used particularly in underdeveloped countries. The limitations of TST were poor specificity in populations with a high prevalence of Bacille Calmette-Guérin (BCG) vaccination and variability of test readers. IGRA is used as an alternative to TST in settings where higher costs can be supported. The lack of studies conducted in high TB incidence countries since previous review, and using relevant assessment tools of the quality appraisal make the need for updated studies and a more comprehensive systematic review. This study aimed to conduct a systematic review of published economic evaluations of screening strategies for LTBI in contacts of TB patients, assess the quality of these studies, and compare the assessment results related to a country’s income level in order to provide information to other countries. The databases were searched in January 2022 including MEDLINE and Scopus. Two independent reviewers evaluated the included studies based on eligibility criteria, data extraction, and quality assessment. Eleven economic evaluations of LTBI diagnostic tests in TB contacts were included. Most studies were conducted in high-income countries (91%) and used cost-effectiveness analysis methods (73%). The quality assessment of reporting and data sources was appropriate, ranging from 71% to 89%. Interventions varied from study to study. The outcomes were cost per life years gained (27%), cost per quality-adjusted life year gained (27%), cost per TB case prevented (36%), and cost per close contact case (10%). In high-income countries which were not countries with high TB burden, the use of IGRA alone for screening TB contacts was cost-effective, whereas TST was cost-effective in only two studies. In comparison to TST, IGRA could reduce false-positive results, resulting in fewer patients undergoing TB treatment and preventive treatment.

## 1. Introduction

Tuberculosis (TB), a communicable disease caused by *Mycobacterium tuberculosis*, is a leading cause of mortality worldwide [1]. It has been reported that 10% of the population infected with *M. tuberculosis* become clinically active TB, while 90% remain in the latent tuberculosis infection (LTBI) phase [2]. The lifetime risk of progression to active TB disease during the first five years ranges from 5% to 10% [3]. Given that the global prevalence of LTBI was 24.8% [4], the World Health Organization (WHO) End TB Strategy aims to reduce the global incidence of TB by 90% by 2035, as compared to 2015 [5]. To achieve the goal of TB eradication, the reservoir of LTBI must be eliminated through identifying and treating cases of LTBI [6].

Either a tuberculin skin test (TST) or interferon-gamma release assay (IGRA) can be used to screen for LTBI [2]. Due to its low cost and minimal infrastructure requirements, TST has been used continuously for decades, particularly in underdeveloped countries [7]. However, in populations with a high prevalence of Bacille Calmette-Guérin (BCG) vaccination, specificity of TST was poor, resulting in false-positive test findings [8]. The limitation of TST was variability of test readers which may lead to misinterpreting test results [8]. In addition, TST requires a cold chain system (2–8 °C), and two visits for injection and interpretation of data [7]. On the other hand, IGRA has been developed and applied for clinical use in several countries [9]. IGRA is used as an alternative to TST in settings that can support higher test acquisition costs [10]. IGRA is considered to be more specific than TST, because T-cells may release interferon-gamma in response to stimulation with *M. tuberculosis*-specific antigens, it is not cross-reactive with BCG, and it does not provide false positive results in the vaccinated population [11]. However, IGRA is more expensive and technically hard to perform than the TST, it requires a laboratory infrastructure [12].

In 2020, the WHO recommends TB preventive treatment for people living with HIV, household contacts of TB patients, and clinical risk groups [13]. However, the number of household contacts receiving preventive treatment was less than 1% in household contacts aged more than 5 years and 20% in children aged less than 5 years [13]. It is highlighted that a significant scale-up of contact tracing is required to reach the targets [13]. Therefore, it is important to identify those persons with confirmed LTBI, mainly among TB contacts, and to implement suitable screening procedures, such as TST and IGRA.

Owing to limited healthcare system resources, the economic evaluation of screening strategies for LTBI in TB contacts is crucial for policymakers to make informed policy decisions. Several economic evaluations of LTBI screening have been undertaken in developed countries, including Switzerland, Germany, France, Canada, Japan, the United Kingdom (UK), the United States (US), Brazil, and South Korea. Although Nienhaus et al. (2011) [14] published a systematic review of cost-effectiveness analysis studies of screening strategies for LTBI in high-risk groups (healthcare workers, immigrants, close contacts), the unavailability of recently published studies especially in TB contacts, the lack of studies in high TB incidence countries since previous systematic review, and the quality appraisal of studies using relevant assessment tools make the need for updating studies and performing a more comprehensive systematic review. Accordingly, the objectives of this study were to conduct a systematic review of published economic evaluations of screening strategies for LTBI in contacts of TB patients, assess the quality of these studies, and compare the assessment results related to a country’s income level in order to provide information to other countries.

## 2. Patients and Methods

This systematic review is conducted following a protocol registered with PROS-PERO (CRD 42022362042) and is reported according to the Preferred Reporting Items for Systematic reviews and Meta-Analyses (PRISMA) 2020 statement: an updated guideline for re-porting systematic reviews [15].

### 2.1. Data Sources and Searches

Up to January 2022, two databases including MEDLINE through PubMed and Scopus databases were searched. Search terms were defined for ‘latent tuberculosis infection’, ‘tuberculosis infection’, ‘contacts’, ‘tuberculin skin test’, ‘interferon gamma release assay’, ‘cost-benefit analysis’, ‘cost-effectiveness analysis’, and ‘cost-utility analysis’. The strategy was divided into three sections. The first section described suitable search terms for LTBI populations and close contacts of TB patients. In the second section, suitable search terms for interventions and comparators were outlined. The last part described suitable search terms for economic studies. The search was limited to the English language, and a similar strategy was used across all databases (see Supplementary Table S1).

### 2.2. Selection of Studies

Two independent reviewers (PY and NC) evaluated the eligible studies based on the information from the title and abstract. If a decision cannot be made based on the abstracts, full articles were reviewed. Disagreement was resolved by consensus and discussion with the third reviewer (UC). Studies were eligible and included if they were (1) studies in participants who had contact with pulmonary TB patients and did not have active TB at age 5 or older, (2) studies comparing TST and IGRA with other interventions i.e., the ELISA-based QuantiFERON Gold (QFT-G) or QuantiFERON Gold In-Tube (QFT-IT) of Cellestis, Carnegie, Australia, or the T-SPOT-based T-SPOT.TB of Oxford Immunotech, Abingdon, UK, and (3) studies in three types of full economic analyses i.e., cost-effectiveness analysis, cost–utility analysis, and cost–benefit analysis that evaluated incremental cost-effectiveness ratio (ICER). Studies published in languages other than English were excluded.

### 2.3. Data Extraction and Quality Assessment

Two independent reviewers (PY and NC) extracted information using standard data extraction forms regarding the research question, methodology, and study characteristics. Data were compared and validated for completeness and accuracy. Three domains of included studies including methodological variations, adequate and transparent reporting, and quality of all input parameters were assessed. Firstly, the reviewers extracted the study characteristics, i.e., the first author affiliation, sources of funding, setting of the study, types of economic evaluation, models used, perspective of study, time horizon, cycle length, discount rate, and sensitivity analysis. Secondly, adequate and transparent reporting of the studies was assessed by the Consolidated Health Economic Evaluation Reporting Standards (CHEERS) statement [15]. Thirdly, the quality of the model input developed by Copper et al. was ranked on a scale from 1 to 6 [16]. The data components including clinical effect sizes, adverse events and complications, baseline clinical data, resource use, costs, and utilities were assessed. The data sources were graded on a scale ranging from 1 to 6, with 1 being the most appropriate data sources. PY and NC (doctoral students) independently evaluated the studies and the data components using the aforementioned tools, respectively. Disagreement was resolved by consensus and discussion with the third reviewer or their supervisor (UC).

### 2.4. Data Synthesis and Analysis

This review compared the cost-effectiveness of several screening strategies for LTBI in TB contacts across studies. The included studies are briefly described. As these studies were conducted from different perspectives, time horizons, settings, interventions/comparators, and outcomes, they cannot be compared. All ICERs and the cost-effectiveness (CE) thresholds were presented as the base of cost analysis. The grouping of included studies associated with income level was obtained from the World Bank classification based on the Gross National Income (GNI) [17]. The year of publication was used in the included studies that did not identify the year of cost analysis.

## 3. Results

### 3.1. Review Profile

**Figure 1:**
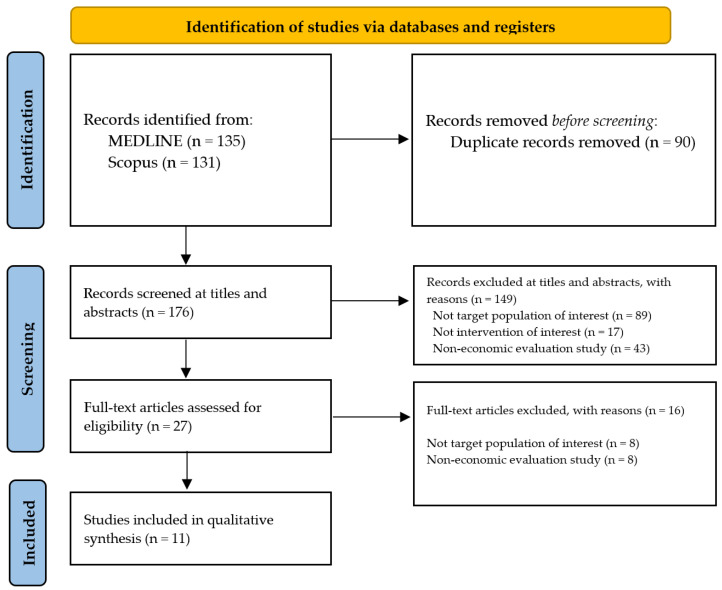
Flow diagram and references of included studies.

The searches conducted in January 2022 uncovered 266 records. After removing duplicates, 176 records were screened for titles and abstracts, in which full texts of 27 potentially relevant articles were reviewed. Of these articles, 16 were excluded, mostly because they did not examine the population of interest (n = 8) or the economic evaluation (n = 8). A total of 11 studies were included in this review [18,19,20,21,22,23,24,25,26,27,28]. The flow diagram is shown in Figure 1.

### 3.2. Study Information

**Figure 2:**
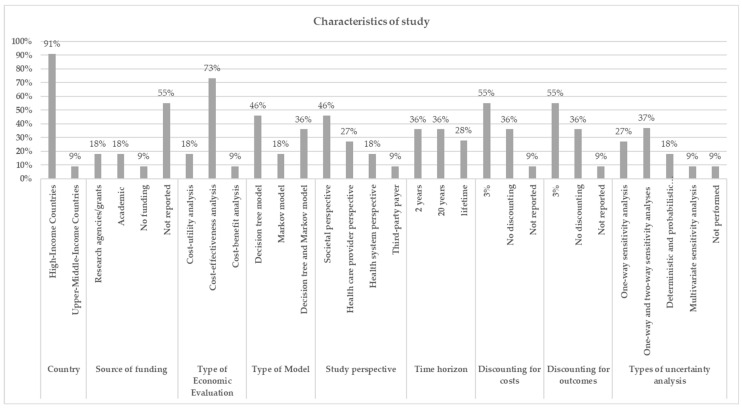
Summary of characteristics and methodologies used in the included studies.

Eleven full economic evaluations of LTBI diagnostic tests (i.e., TST, IGRA, and no testing) were published between 2007 and 2018 (Table 1). As shown in Figure 2, most studies (10 studies, 90.9%) were conducted in high-income countries (HICs) [18,20,21,22,23,24,25,26,27,28], while only one study was undertaken in an upper-middle-income country (UMIC) [19]. The majority of the included studies (8 studies, 72.7%) were cost-effectiveness analysis (CEA) studies [18,19,20,21,22,26,27,28], whereas two studies (18.2%) were cost-utility analysis (CUA) studies [24,25], and one study (9.1%) was cost-benefit analysis (CBA) [23]. The following perspectives, i.e., societal (5 studies, 45.5%) [23,25,26,27,28], healthcare provider (3 studies, 27.3%) [20,21,22], health system (2 studies, 18.2%) [18,19], and third-party payer (1 study, 9.1%) were adopted [24]. Decision tree and Markov models (4 studies, 36.4%) [24,25,27,28], Markov models (2 studies, 18.2%) [20,26], and decision tree models (5 studies, 45.5%) [18,19,21,22,23] were applied. There were studies with time horizons of 2 years (4 studies, 36.4%) [18,19,21,23], 20 years (4 studies, 36.4%) [24,26,27,28], and lifetime (3 studies, 27.3%) [20,22,25]. Six studies (54.5%) utilized a 3% discount rate applied for adjusting future costs and outcomes to their present values [20,22,24,25,27,28], whereas four studies did not use discount rate [18,19,21,23], and one study did not report discount rate [26]. For uncertainty analysis, one-way (3 studies, 27.3%) [21,22,28], both one-way and two-way (4 studies, 36.4%) [18,19,20,24], deterministic and probabilistic (2 studies, 9.1%) [23,25], multivariate sensitivity analysis (1 study, 9.1%) [27], and one study (9.1%) [26] did not perform sensitivity analysis.

### 3.3. Quality Assessment of Reporting

**Figure 3:**
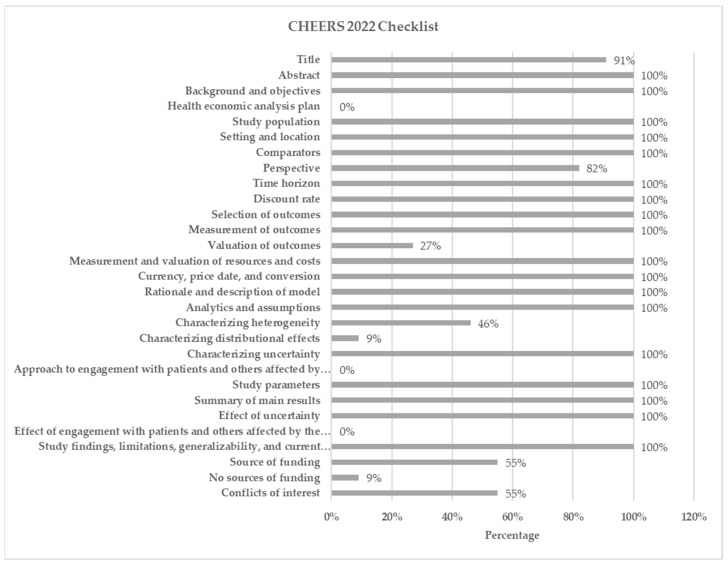
Quality of reporting results based on the 2022 CHEERS checklist.

The quality assessment results of reporting based on the 2022 CHEERS checklist are shown in Figure 3 (see Supplementary Table S2). Of all 28 domains in the CHEERS 2022 checklist, 18 items were reported by all studies. However, no study disclosed the strategy for health economic analysis, the approach to engaging patients and others affected by the study, or the effect of engaging patients and others affected by the study. Title (91%), perspective (82%), evaluation of outcomes (27%), characterization of heterogeneity (46%), and characterization of distributional effects (9%) were not provided in all studies. Six studies (55%) did not report funding source and one study (9%) did not have a source of funding. Moreover, six studies (55%) did not declare conflicts of interest.

### 3.4. Quality Assessment of Input Data Sources

**Figure 4:**
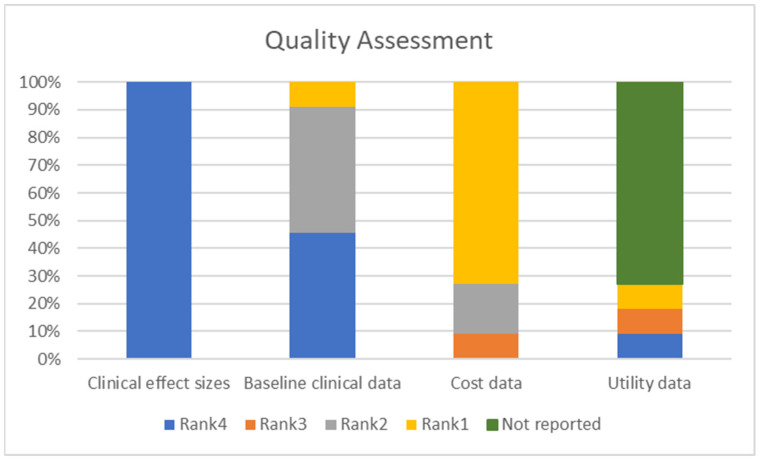
Quality assessment of evidence used in economic evaluation studies. Y-axis represents a rank based on the quality assessment (%). X-axis represents for data components.

The quality of input data used to estimate parameters for the models was evaluated using the potential hierarchies of evidence. The list of sources of input data is clinical effect sizes, baseline clinical data, resource use, costs, and utilities. Sources are ranked from 1 to 6, the most appropriate source set a rank of 1 [16]. The baseline clinical data (LTBI reactivation) of five studies (Rank = 2; 45%) were derived from analysis of administrative databases in population of interest [23,25,26,27,28] and other five studies (Rank = 4; 45%) were derived from randomized controlled trial (RCT) [18,19,20,21,22], while only one study (Rank = 1; 10%) used country-specific databases [24]. Correspondingly, the clinical effect sizes for the sensitivity and specificity of LTBI testing data were derived from cohort studies for all studies (Rank = 4; 100%) [18,19,20,21,22,23,24,25,26,27,28]. Eight studies (Rank = 1; 73%) used costing parameters generated from local data sources [18,19,20,21,22,23,24,25], while two studies (Rank = 2; 18%) were derived from published cost studies [27,28], and one study (Rank = 3; 9%) used a previous economic evaluation study [26]. However, only three studies reported utility parameters, one study (Rank = 4; 9%) referred from unclearly utility data [25], one study (Rank = 3; 9%) from a previous study [20], and another study (Rank = 1; 9%) employed direct utility assessment from the disease of interest [24]. The quality assessment results of input data sources using a tool developed by Cooper et al. are summarized in Figure 4 [16].

### 3.5. Cost-Effectiveness Analysis Results

The outcomes were quantified by cost per life years (LY) gained (3 studies, 27%) [22,27,28], cost per quality-adjusted life year (QALY) gained (3 studies, 27%) [20,24,25], cost per TB case prevented (4 studies, 36%) [18,19,21,26], and cost per close contact case, i.e., the cost for laboratory investigation LTBI treatment per close contact (1 study, 9%) [23]. Five studies (45%) provided their country cost-effectiveness thresholds [20,24,25,27,28], while six studies (55%) did not report their thresholds [18,19,21,22,23,26]. Moreover, among studies conducted in France, Canada, Japan, Germany (2009), and the US indicated that the use of IGRA alone was cost-effective [20,22,23,24,25]. However, the studies conducted in the UK and Germany (2007) showed that using IGRA for only TST-positive cases (IGRA as a confirmatory test) was cost-effective [21,28]. According to the studies conducted in Switzerland and Canada, IGRA or IGRA as a confirmatory test was cost-effective, but TST alone or IGRA alone was cost-saving, respectively [26,27]. In contrast to the studies in Brazil and South Korea, TST alone was the most cost-effective strategy [18,19] (Table 2).

## 4. Discussion

To the best of our knowledge, this systematic review is the first to identify and update studies of economic evaluation on screening strategy for LTBI in contacts of TB patients since the last search in 2011 [14], as well as to assess the quality of the studies based on the methodology used according to the CHEER checklist [15] and data sources by Cooper et al. [16]. Although a systematic review of cost-effectiveness analysis studies of screening strategies for LTBI in high-risk groups, e.g., healthcare workers, immigrants from high-incidence countries, and close contacts, has been previously published, the quality of these studies has not been evaluated, yet [14]. This systematic review focused on TB contacts for whom the worldwide diagnosis and treatment for LTBI was minimal. To achieve the target of ending TB by 2030, a significant scale-up of contact tracing will be needed. Consequently, it is crucial to identify contacts of TB patients with confirmed LTBI and to implement appropriate screening strategies. For this reason, our systematic review focused on the economic evaluation of screening strategies for LTBI among contacts of TB patients. In this systematic review, 11 studies were included, four studies of which were updated and compared to the previous systematic review [14].

There were numerous studies conducted cost-effectiveness analysis using a decision tree model to evaluate cost and outcomes [18,19,21,22,27,28]. This model was appropriate because it can capture the cost and outcomes of LTBI screening in a short period of time. Approximately half of the studies were conducted from a societal perspective, which covered all costs incurred by everyone in the society, i.e., direct medical, direct non-medical, and indirect costs [23,25,26,27,28], while six studies were conducted from the perspectives of healthcare provider, health systems, and third-party payers, which covered only direct medical costs and provided policymakers with guidance on its coverage [18,19,20,21,22]. Remarkably, the most crucial cost of all studies was active TB treatment, which covered only direct medical costs.

In addition, it is noted that there may be a difference in clinical practice when using preventive treatment regimens for LTBI, as these treatment regimens are currently available. In all included studies, the standard preventive treatment is isoniazid for 6 to 9 months of continuous treatment [18,19,20,21,22], which raises concerns regarding side effects and treatment length. Recently, short course regimens, e.g., three months of once-weekly isoniazid plus rifapentine (3HP), four months of daily rifampin (4R), and three months of daily isoniazid plus rifampin (3HR), are preferentially recommended over isoniazid monotherapy [29] due to fewer adverse events and higher adherence. However, these short-course regimens are more expensive, and this may have an impact on the cost-effectiveness results of the LTBI screening strategy.

The vast majority of included studies were conducted in HICs, which were evidently not countries with high TB burden where the WHO strongly recommends screening for LTBI [2]. In contrast, just only one economic evaluation was conducted in Brazil, a UMIC with a high TB burden [19]. The results suggested that screening for LTBI was not a high priority in these countries and that implementation of WHO LTBI guidelines was hindered by product shortages (i.e., TST) and high costs (i.e., IGRAs). Thus, in the context of UMICs with a high TB burden, clinical practice guidelines and accessible screening techniques may vary from those of HICs. It was suggested that future studies should be further investigated on the cost-effectiveness of the screening strategy for LTBI among contacts of TB patients in the area with the highest prevalence, i.e., South-East Asia, accounting for 35% of the entire global burden of LTBI [4].

Moreover, our systematic reviews revealed that the quality assessment of reporting and data sources was appropriate. The rating range for the transparency of reporting was fairly large, ranging from 71% to 89%. Although the extent of reporting based on the CHEERS 2022 statement has been already announced [15], all studies were published prior to 2022. This might be due to the fact that they were conducted in HICs where expertise in economic evaluation was recognized. Given that the majority of studies were conducted in HICs where cost databases were available, it is noted that cost data were obtained from highly rated sources. In the context of baseline clinical data, half of the studies were estimated from RCT, because they were conducted in settings with a low incidence of TB and low LTBI reactivation cases. The clinical effect sizes data pertaining to the sensitivity and specificity of LTBI testing, which all studies were derived from cohort studies because there might be a lack of RCT studies. Moreover, only three studies reported QALYs as an outcome, these suggested that this outcome might not distinguish between test performance.

The diagnostic tests were included in this analysis as recommended by the WHO 2018 guidelines. The testing criteria recommended that either TST or IGRA might be used for LTBI screening [2]. Results from our systematic review uncovered that IGRA was cost-effective in HICs including Germany [23,28], Switzerland [27], Canada [24], Japan [25], France [22], the US [20], and the UK [21], whereas TST was cost-effective in Brazil [19] and South Korea [18]. Similar to a previously published systematic review [14], this review revealed that the use of IGRA was cost-effective given that IGRA might reduce false-positive results, resulting in fewer patients undergoing TB treatment and preventive treatment when compared with TST. In spite of this, there was a considerable disparity in the findings that TST was cost-effective in Brazil [19] and South Korea [18]. This can be explained that despite a higher number of preventative therapies for LTBI, TB treatment, adverse events, and healthcare costs in Brazil [19] and South Korea [18] are considerably lower. These lower costs had a substantial influence on the ultimate outcomes. In addition, IGRA, a confirmatory test for TST positive, was cost-effective in Germany [23,28], Switzerland [27], and the UK [21]. The results of these studies can be explained that IGRA was more expensive and more accurate. Consequently, there are fewer contact cases that need preventative treatment when IGRA is used as a confirmatory test. Nonetheless, the benefit of IGRA cannot offset the reduced incremental costs associated with IGRA as a confirmatory test, which is more cost-effective than IGRA alone.

Although this systematic review included more up-to-date research than a previous review and used the most recent version of the CHEERS 2022 checklist for evaluating standardization and transparency of reporting, some limitations need to be taken into consideration. First, LY gained, QALY gained, cost per TB case avoided, and cost per close contact case were measured differently among studies, making it impossible to compare across studies. Second, most included studies were conducted in HICs, except for Brazil. During 2016–2020, the WHO mentioned just two studies in countries with a high TB burden, namely Brazil [19] and South Korea [18]. Therefore, it is highlighted that the majority of included studies were from HICs and not from countries with a high TB burden. Thirdly, this systematic review excluded unpublished studies possibly conducted in low- and middle-income countries (LMICs) and low-income countries (LICs).

## 5. Conclusions

This updated review of studies on the economic evaluation of screening strategy for LTBI in contacts of TB patients indicated that the use of either IGRA alone or IGRA as a confirmatory test for a positive TST was cost-effective in HICs such as Germany, Switzerland, Canada, Japan, France, the US, and the UK. In addition to this, TST alone proved economical in Brazil and South Korea.

## Figures and Tables

**Table 1:** The characteristics of economic evaluation studies.

Authors	Country	Types of EE	Intervention/Comparator	Perspective	Time Horizon	Modelling	Discount Rate (%)	Sensitivity Analysis
Diel et al. (2007) [27]	Switzerland	CEA	1. TST ≤ 5 mm, 2. TST ≤ 10 mm 3. TST ≤ 15 mm 4. T-SPOT.TB, 5. TST ≤ 10 mm/T-SPOT.TB	The Swiss social	20 years	Decision tree, Markov	3%	Multivariate
Diel et al. (2007) [28]	Germany	CEA	1. QFT-G, 2. TST > 5 mm, 3. TST > 10 mm, 4. TST > 5 mm/QFT	Societal	20 years	Decision tree, Markov	3%	One-way
Oxlade et al. (2007) [26]	Canada	CEA	1. No screening, 2. TST, 3. QFT	Societal	20 years	Markov	No report	No
Marra et al. (2008) [24]	Canada	CUA	1. QFT-G, 2. TST/QFT-G, 3. TST	The third-party payer	20 years	Decision tree, Markov	3%	One-way and two-way
Kowada et al. (2008) [25]	Japan	CUA	1. QFT, 2. TST/QFT, 3. TST	Societal	lifetime	Decision tree, Markov	3%	One-way probabilistic
Diel et al. (2009) [23]	Germany	CBA	1. QFT 2. TST, 3. TST/QFT	Societal	2 years	Decision tree	No	Deterministic probabilistic
Deuffic-Burban et al. (2010) [22]	France	CEA	1. TST ≤ 10 mm, 2. QFT, 3. TST/QFT 4. No testing	The French health care payers’	Lifetime	Decision tree	3%	One-way
Pooran et al. (2010) [21]	UK	CEA	1. TST, 2. T-SPOT.TB, 3. TST/T-SPOT.TB, 4. QFT-GIT, 5. TST/QFT-GIT	A UK healthcare	2 years	Decision tree	No	Univariate deterministic
Linas et al. (2011) [20]	US	CEA	1. No screening, 2. TST, 3. IGRA	Healthcare provider	Lifetime	Markov	3%	one-way and two-way
Steffen et al. (2013) [19]	Brazil	CEA	1. QFT-GIT, 2. TST/QFT-GIT, 3. TST	The National Health System	2 years	Decision tree	No	One-way and two-way
Sohn et al. (2018) [18]	South Korea	CEA	1. QFT-GIT, 2. TST, 3. TST/QFT-GIT	The health system	2 years	Decision tree	No	One-way and two-way

Tuberculin skin test = TST; QuantiFERON-TB = QFT; QuantiFERON-TB Gold = QFT-G; QuantiFERON-TB Gold In-Tube = QFT-GIT; TST/QFT = TST followed by a QFT; TST/QFT-G = TST followed by a QFT-G; TST/QFT-GIT = TST followed by a QFT-GIT; Economic analyses = EE.

**Table 2:** Summary of cost-effectiveness analysis results.

Study	Country	Intervention	Result	Reported ICER at Base Year	Base Year	CE Threshold
Diel et al. (2007) [28]	Germany	TST/QFT with LTBI treatment vs. TST/QFT with no LTBI treatment	TST/QFT is cost-effective in reducing the TB burden	ICER = TST/QFT with non-treatment dominated ($/LYG)	US dollars; 2004	USD 50,000 per LYG
Diel et al. (2007) [27]	Switzerland	T-SPOT.TB, TST/T-SPOT.TB with LTBI treatment vs. T-SPOT.TB, TST/T-SPOT.TB with no LTBI treatment	T-SPOT.TB or TST/T-SPOT.TB is cost-effective in reducing the TB burden	ICER (20 yrs) = €11,621 per LYG	Euros; 2004	EUR 40,195 per LYG
		T-SPOT.TB, TST/T-SPOT.TB with LTBI treatment vs. T-SPOT.TB, TST/T-SPOT.TB with no LTBI treatment	T-SPOT.TB or TST/T-SPOT.TB is cost-effective in reducing the TB burden	ICER (40 yrs) = €23,692 per LYG	Euros; 2004	EUR 40,195 per LYG
Oxlade et al. (2007) [26]	Canada	TST, QFT vs. no screen	TST or QFT would be cost saving	ICER = CA$ 23,330 per case prevented (TST), 20,737 per case prevented (QFT)	Canadian dollars; 2004	Not reported
Marra et al. (2008) [24]	Canada	QFT-G in BCG-positive contacts, TST for others vs. TST in all contacts	QFT-G in BCG-positive contacts was dominant	ICER = QFT-G in BCG-positive contacts was dominant ($CA/QALY)	Canadian dollars; 2005	CAD 50,000 to gain an additional QALY
Kowada et al. (2008) [25]	Japan	QFT vs. TST/QFT vs. TST	QFT-alone strategy was dominant	ICER = QFT was dominant ($US/QALYs)	Japanese Yen; 2007	JPY 25,000 /QALY gained
Diel et al. (2009) [23]	Germany	QFT	The QFT assay alone generates less cost and decreases more TB cases.	Cost = EUR 215.79 per close contact	Euros; 2008	Not reported
Deuffi c-Burban et al. (2010) [22]	France	QFT vs. TST/QFT	QFT is more effective and cost-effective than TST/QFT	ICER = EUR 730 per LYG	Euros; 2007	Not reported
Pooran et al. (2010) [21]	UK	TST/T-SPOT.TB vs. no screening	TST/T-SPOT.TB and TST/QFT-GIT are cost effective	ICER = £37,206 per active case prevented	GBP; 2008	Not reported
Linas et al. (2011) [20]	USA	IGRA vs. TST	IGRA screening was more cost effectivethan TST screening.	ICER = $21,500 /QALY	US dollars; 2011	USD 50,000 per QALY gained, $100,000 per QALY gained
Steffen et al. (2013) [19]	Brazil	TST strategy	TST was the most cost-effective strategy for averting new TB cases	US$ 16,021/averted case for TST strategy.	US dollars; 2010	Not reported
Sohn et al. (2018) [18]	South Korea	QFT-GIT vs. TST	TST was cost-effective	ICER = US$ 140,933/averted case	US dollars; 2015	Not reported

Tuberculin skin test = TST; QuantiFERON-TB = QFT; QuantiFERON-TB Gold = QFT-G; QuantiFERON-TB Gold In-Tube = QFT-GIT; TST/QFT = TST followed by a QFT; TST/QFT-G = TST followed by a QFT-G; TST/QFT-GIT = TST followed by a QFT-GIT.

## Data Availability

All data underlying the results are available as part of the article and no additional source data are required.

## Supplementary Materials

The following supporting information can be downloaded at: https://www.mdpi.com/article/10.3390/ijerph192013529/s1, Table S1: Search strategies; Table S2: The CHEERS checklist resulted in scores.
