# Supplementary material for: Economic Evaluation of Screening Strategy for Latent Tuberculosis Infection (LTBI) in Contacts of Tuberculosis Patients: Systematic Review and Quality Assessment
Source: Int J Environ Res Public Health. 2022 Oct 19;19(20):13529. doi: 10.3390/ijerph192013529 (PMC9603136; doi:10.3390/ijerph192013529)
Supplement: Supplementary file 1 [file ijerph-19-13529-s001.zip › ijerph-1955581-supplementary.pdf]

Supplementary

Table S1 Search strategies

Database: **Pubmed**; Jan 2022

Number	Query	Results
1	Tuberculo*	283,658
2	tb	78,404
3	laten* tubercul*	8,947
4	ltbi	8,474
5	contact*	457,898
6	or/1-4	314,626
7	6 AND 5	7,901
8	tuberculin skin test	17,201
9	TST	6,513
10	interferon gamma release assay	4,658
11	igra*	1,972
12	ifn-gamma	86,556
13	qunatiferon tb gold	2
14	qft-g	196
15	t-spot	729
16	(qunatiferon tb gold) OR (qft-g)	198
17	(ifn-gamma) OR (qunatiferon tb gold)	86,557
18	or/8-17	109,892
19	Economic*	814,624
20	Cost*	912,085
21	Cost-benefit	119,202
22	Cost-effectiveness	142,557
23	Cost-utility	5,585
24	or/19-23	1,452,154
25	6 AND 5 AND 18 AND 24	151
28	Filters; English	135

Database: **Scopus**; Jan 2022

Number	Query	Results
1	TITLE-ABS-KEY (tuberculo*)	364,058
2	TITLE-ABS-KEY (tb)	105,065
3	TITLE-ABS-KEY (laten* AND tubercul*)	11,411
4	TITLE-ABS-KEY (ltbi)	2,832
5	TITLE-ABS-KEY (contact*)	1,289,822
6	TITLE-ABS-KEY (tuberculin AND skin AND test)	11,166
7	TITLE-ABS-KEY (tst)	9,657
8	TITLE-ABS-KEY (interferon AND gamma AND release AND assay)	13,590
9	TITLE-ABS-KEY (igra*)	2,429
10	TITLE-ABS-KEY (ifn-gamma)	87,697
11	TITLE-ABS-KEY ('qunatiferon AND tb AND gold')	2
12	TITLE-ABS-KEY (qft-g)	219
13	TITLE-ABS-KEY (t-spot)	1,038
14	TITLE-ABS-KEY (economic*)	2,477,042
15	TITLE-ABS-KEY (cost*)	3,241,840
16	TITLE-ABS-KEY ('cost-benefit')	216,563
17	TITLE-ABS-KEY ('cost-effectiveness')	326,184
18	TITLE-ABS-KEY ('cost-utility')	10,937
19	or/1-4 AND 5	9,603
20	or/6-13	113,759
21	or/14-18	5,126,860
22	19 AND 20 AND 21	150
23	Filters; English	131

Table S2 The CHEERS checklist resulted in scores

	**CHEERS 2022 checklist**	**Number of studies**	%	Diel et al. (2007) Germany	Diel et al. (2007)	Deuffi c-Burban et al. (2010)	Marra et al. (2008)	Kowada et al. (2008)	Steffen et al. (2013)	Sohn et al. (2018)	Oxlade et al. (2007)	Diel et al. (2009)	Pooran (2010)	Linas (2011)
1	Title	10	90.9	1424	321	471	1414	235	1	496	16	1838	1	Not report
	**Abstract**													
2	Abstract	11	100.0	1424	321	471	1414	235	1	496	16	1838	1	590
	**Introduction**													
3	Background and objectives	11	100.0	1424-1425	321-322	471	1414	235-236	1	496	16	1839	2	590
	**Methods**													
4	Health economic analysis plan	0	0.0	Not report	Not report	Not report	Not report	Not report	Not report	Not report	Not report	Not report	Not report	Not report
5	Study population	11	100.0	1425	322	473	1414-1415	236	2	497	16	1839	2	591
6	Setting and location	11	100.0	1425	321-322	473	1414-1415	236	2	497	16	1839	2	591
7	Comparators	11	100.0	1425	322	473	1415	236	2	497	18-19	1839	2	591
8	Perspective	9	81.8	1427	325	473	1418	242	2	496	Not report	1850	2	Not report
9	Time horizon	11	100.0	1424	322	473	1415	236	2	497	18	1839	2	593
10	Discount rate	11	100.0	1428	327	473	1419	242	2	500	18	1849	2	591
11	Selection of outcomes	11	100.0	1428	327	473	1415	237-239	2	497	18	1848	2	591
12	Measurement of outcomes	11	100.0	1426	324	473	1415-1418	237-239	2	497	18	1848	2	591-593
13	Valuation of outcomes	3	27.3	Not report	Not report	Not report	1419	239	Not report	Not report	Not report	Not report	Not report	593

	CHEERS 2022 checklist	Number of studies	%	Diel et al. (2007) Germany	Diel et al. (2007)	Deuffi c-Burban et al. (2010)	Marra et al. (2008)	Kowada et al. (2008)	Steffen et al. (2013)	Sohn et al. (2018)	Oxlade et al. (2007)	Diel et al. (2009)	Pooran (2010)	Linas (2011)
14	Measurement and valuation of resources and costs	11	100.0	1427-1428	325	473	1418	241	2	497-500	19	1839-1844	6	593
15	Currency, price date, and conversion	11	100.0	1427	325	473	1419	241	2	500	19	1849	2	593
16	Rationale and description of model	11	100.0	1426	322-323	472-473	1414-1415,1416	238-245	2	498	18	1839-1846	2,3	591-593
17	Analytics and assumptions	11	100.0	1426 & 1429	322-327	473-474	1415-1418	237-238,244-245	2	497	17	1839-1847	2,4	591
18	Characterizing heterogeneity	5	45.5	Not report	Not report	476	1420	Not report	Not report	Not report	17	Not report	2	591-592
19	Characterizing distributional effects	1	9.1	Not report	Not report	Not report	1419-1420	Not report	Not report	Not report	Not report	Not report	Not report	Not report
20	Characterizing uncertainty	11	100.0	1429	327	475	1420	248-249	5	500	20	1844 and 1847	5	593
21	Approach to engagement with patients and others affected by the study	0	0.0	Not report	Not report	Not report	Not report	Not report	Not report	Not report	Not report	Not report	Not report	Not report
	**Results**													
22	Study parameters	11	100.0	1428	322,324,326	473-474	1417	246	3	499	17	1841	6	592
23	Summary of main results	11	100.0	1430	329	475	1420	250	5	500	20	1844-1847	7	594-595

	CHEERS 2022 checklist	Number of studies	%	Diel et al. (2007) Germany	Diel et al. (2007)	Deuffi c-Burban et al. (2010)	Marra et al. (2008)	Kowada et al. (2008)	Steffen et al. (2013)	Sohn et al. (2018)	Oxlade et al. (2007)	Diel et al. (2009)	Pooran (2010)	Linas (2011)
24	Effect of uncertainty	11	100.0	1429	327	475	1421	242	5	501	20	1844 and 1847	5	595-596
25	Effect of engagement with patients and others affected by the study	0	0.0	Not report	Not report	Not report	Not report	Not report	Not report	Not report	Not report	Not report	Not report	Not report
	**Discussion**													
26	Study findings, limitations, generalizability, and current knowledge	11	100.0	1432-1433	327-331	476-477	1420-1422	247-250	5	501-502	22	1847-1848	9	595-598
	**Other relevant information**													
27	Source of funding	6	54.5	Not report	Not report	477	1422	250	1	502	Not report	Not report	Not report	590
28	Conflicts of interest	6	54.5	1425	Not report	477	1422	250	Not report	502	Not report	1852	Not report	Not report
